# A novel analysis strategy for integrating methylation and expression data reveals core pathways for thyroid cancer aetiology

**DOI:** 10.1186/1471-2164-16-S12-S7

**Published:** 2015-12-09

**Authors:** Bugra Ozer, Osman Uğur Sezerman

**Affiliations:** 1 Biological Sciences and Bioengineering Program, Faculty of Engineering and Natural Sciences, Sabanci University, Istanbul, Turkey; 2 Advanced Genomics and Bioinformatics Research Center (IGBAM), The Scientific and Technological Research Council of Turkey (TUBITAK), 41470, Gebze, Kocaeli, Turkey; 3 Department of Biostatistics and Medical Informatics, Faculty of Medicine, Acıbadem University, Ataşehir, Istanbul, 34752, Turkey

## Abstract

**Background:**

Recently, a wide range of diseases have been associated with changes in DNA methylation levels, which play a vital role in gene expression regulation. With ongoing developments in technology, attempts to understand disease mechanism have benefited greatly from epigenetics and transcriptomics studies. In this work, we have used expression and methylation data of thyroid carcinoma as a case study and explored how to optimally incorporate expression and methylation information into the disease study when both data are available. Moreover, we have also investigated whether there are important post-translational modifiers which could drive critical insights on thyroid cancer genetics.

**Results:**

In this study, we have conducted a threshold analysis for varying methylation levels to identify whether setting a methylation level threshold increases the performance of functional enrichment. Moreover, in order to decide on best-performing analysis strategy, we have performed data integration analysis including comparison of 10 different analysis strategies. As a result, combining methylation with expression and using genes with more than 15% methylation change led to optimal detection rate of thyroid-cancer associated pathways in top 20 functional enrichment results. Furthermore, pooling the data from different experiments increased analysis confidence by improving the data range. Consequently, we have identified 207 transcription factors and 245 post-translational modifiers with more than 15% methylation change which may be important in understanding underlying mechanisms of thyroid cancer.

**Conclusion:**

While only expression or only methylation information would not reveal both primary and secondary mechanisms involved in disease state, combining expression and methylation led to a better detection of thyroid cancer-related genes and pathways that are found in the recent literature. Moreover, focusing on genes that have certain level of methylation change improved the functional enrichment results, revealing the core pathways involved in disease development such as; endocytosis, apoptosis, glutamatergic synapse, MAPK, ErbB, TGF-beta and Toll-like receptor pathways. Overall, in addition to novel analysis framework, our study reveals important thyroid-cancer related mechanisms, secondary molecular alterations and contributes to better knowledge of thyroid cancer aetiology.

## Introduction

Most common endocrine cancer observed in follicular cells is the Human Papillary Thyroid Cancer. It has highest incident rate among endocrine cancers, and it occurs in all age groups from children to older adults.

Biology of thyroid cancer includes both genetic and epigenetic alterations as driving forces of the disease state [1]. In literature, certain precursor genes have been associated with Human Thyroid Cancer. RAS gene mutations have been detected in 5-20% and BRAF gene mutations have been reported in 28-69% of papillary thyroid cancer cases [2,3]. Variations in RET gene have also been frequently observed in papillary thyroid cancer cases [4,5]. Additionally, there are several genes reported in the work of Cancer Genome Atlas Research for Papillary Thyroid Carcinoma such as; PPARG, ALK, NTRK3 [6].

In addition to the studies on investigating genetic reasons behind thyroid cancer, various studies have been conducted to understand epigenetic alterations in thyroid cancer. In papillary thyroid cancer, numerous methylation studies have revealed that *RARB (Retionoic Acid Receptor), CDKN2A (Cyclin-Dependent Kinase Inhibitor 2A), TSHR (Thyroid Stimulating Hormone Receptor), CDH1 (Cadherin 1, type 1), DAPK (Death-Associated Protein Kinase 1), MLH1 (mutL Homolog 1) and RASSF1A(Ras associated gene) *are observed to have significantly altered methylation levels [7,8]. Specifically RAS-MAPK signal activation via *RASSF1A *methylation has been detected in 20% of papillary thyroid cancer cases [9]. Additionally, tumour suppressors and oncogenes such as *KISS1R, ADAMTS5, HOXB4, TCL1B, NOTCH4, TIMP3 *can also be added to previous gene list of differentially methylated genes that have been observed in various disease conditions [10].

Besides individual genes, some signalling pathways are also reported to be affected with thyroid cancer such as; *MAPK Signalling Pathway, the Natural Killer Cell pathway*, *The HIF1α pathway *and *Thyroid-stimulating hormone receptor pathway *[1]. Additionally, *Toll-like receptor signalling pathway *[11], *Pentose-phosphate pathway *[12] and *ErbB pathway *(*Mtor pathway*) have previously been linked with thyroid cancer [7]. Other pathways such as; *TGF-beta signalling pathway *[13], *VEGF signalling pathway *[14], *Neurotrophin signalling pathway *[15], *Focal adhesion *[16], *Extracellular matrix activity *[17], *Adherens junction *[18], *p53 signalling pathway *[19], *Notch signalling pathway *[20] are described as being active at thyroid cancer pathogenesis. Also observed at other cancer types, *Apoptosis, Fc epsilon RI signalling pathway, Leukocyte transendothelial migration, T cell receptor signalling pathway, B cell receptor signalling pathway, GnRH signalling pathway *and *Transcriptional misregulation in cancer *are shown as being involved in thyroid cancer as well [21-23]. Overall, even though there are several reported genes and pathways that are linked with thyroid cancer, mechanisms employed in the disease development still remain unclear.

In recent decades, the nature of DNA methylation became a hot research topic with ongoing developments in technology. There are concrete evidences about epigenetics that, it plays a crucial role in disease development, especially in cancer [24-26]. From this perspective, incorporating epigenetic information into disease identification studies would shed light on the disease aetiology, thus improving the treatment procedure. For this purpose, a highly preferred way is to conduct both expression and methylation experiments. However, integrating methylation and expression data is a problem that is commonly confronted due to the complex relationship between methylation and expression. Recent studies show that searching for correlation between methylation and expression data is the most adopted strategy on tackling this problem. In this type of analysis, statistical analysis of both methylation and expression data are conducted separately and at the final stage, these results are compared with each other [27-32]. Another approach is to merge methylation and expression data prior to any kind of analysis by implementing general data integration algorithms [33,34]. Although general data integration algorithms enable merging of multi-layered data in an efficient way, they do not yield optimal results as they omit the nature of biological relationship between methylation and expression.

Methylation is a way to regulate the gene expression level mediated by environmental factors as well as post-translational modifications and noncoding RNAs [35].The biological relation between methylation and gene expression is believed to be so that, for most of the genes, methylation has crucial role in repressing gene expression by blocking the promoters at which transcription factors can bind and initiate the expression process. Thus, it is expected to observe an inverse correlation between expression and methylation. However, there are also other works showing that there is not always inverse correlation between methylation and expression, hence transcription is defined as being independent of methylation for some of the genes [36].

Experimental results show that a change in methylation level does not always lead to a corresponding change in expression level due to variety of factors. At this point, the definition of a certain threshold considering the high correlation between methylation and expression may be beneficial when both methylation and expression data are available. In microarray expression experiments, a simple fold change of 2 is recommended between two conditions to obtain more reliable results. In methylation experiments, Beta-value (β) is defined as the ratio of methylated probe intensity over the overall intensity composed of methylated and unmethylated probes. However, delta beta (Δβ) threshold is not well defined in the publications. The question "whether methylation significance values always correspond to significant alteration in methylation level", remains unanswered. In this sense, B-Value threshold in methylation experiments is an issue that needs to be seriously addressed; hence setting a valid threshold for methylation change between two conditions may be helpful in obtaining more accurate list of significantly methylated or unmethylated genes.

In this study, we have investigated how to obtain optimal results when both expression and methylation information are available and we have tried to understand the interplay between methylation and expression in thyroid cancer. For this purpose, our research focused on two main topics; whether setting a methylation level threshold improves the outcome of the analysis and how to obtain optimal results when expression and methylation information are both available. In this sense, we have conducted a threshold analysis for varying methylation levels considering the inverse correlation between methylation and expression. Similarly, in order to further understand whether using expression or methylation reveals more information about disease aetiology, we have made comparisons between 4 different datasets and 10 different analysis strategies. To support our findings and to show generalizability, we have also applied our framework to independent thyroid cancer dataset. Overall, in addition to a novel analysis framework, our study reveals potentially important thyroid-cancer related mechanisms and secondary molecular alterations which can contribute to better understanding of thyroid cancer aetiology.

## Methods

### Dataset

Dataset consisting of 8 normal and 10 tumour samples are obtained from Batch230 and dataset consisting of 6 normal and 6 tumour samples are obtained from Batch250 Thyroid Cancer Carcinoma data in The Cancer Genome Atlas (TCGA) [6]. This dataset was used as a case study and training dataset. Additionally, we have also downloaded another 30 samples from the same source to test our findings on another independent dataset. In TCGA, while selecting normal tissue samples we have focused on including samples which are "matched" to the anatomic site of tumour. In correlation, while selecting tumour samples we have carefully chosen samples which have "matched" normal samples included in the same experiment.

We have only selected the samples that contain both RNA sequencing and methylation data at our study. According to data providers, all methylation data was obtained from Illumina Human Methylation 450k Chip, whereas all RNA sequencing data was obtained from Illumina HiSeq machine. Data consisting of intensity values corresponding to each region for methylation chip and counting values corresponding to each gene for RNA-Seq are downloaded for our study.

For both methylation and RNA sequencing (RNA-Seq) experiments, statistical analyses are conducted for each batch independently and also by pooling both batches together before pre-processing the data.

### Methylation analysis

Methylation is not a gene-specific but a region-specific phenomenon. Methylation occurring at different gene regions may end up having different outcomes. In our methylation analysis, we have investigated methylations occurring in first exon, 3'UTR, 5'UTR, gene body, intergenic region and transcription start sites using ChAMP package [37] which is available in R. ChAMP pipeline is specifically designed for analysis of Illumina HumanMethylation450k chip and it involves a sliding window approach (Probe Lasso) for annotating CpG regions with genomic locations [38].

In array-based methylation experiments, both Beta-value and M-value statistics are used as metrics to measure methylation levels. Beta-Value in methylation experiments is the estimate of methylation level using the ratio of the methylation probe intensity and the overall intensity whereas M-value is a logit transformation of Beta-Value. For easier functional interpretation of the results, we have used Beta-Value at our analysis, which provides more intuitive biological interpretation as it roughly corresponds to the percentage of a methylation on a specific site [39].

**Figure 1:**
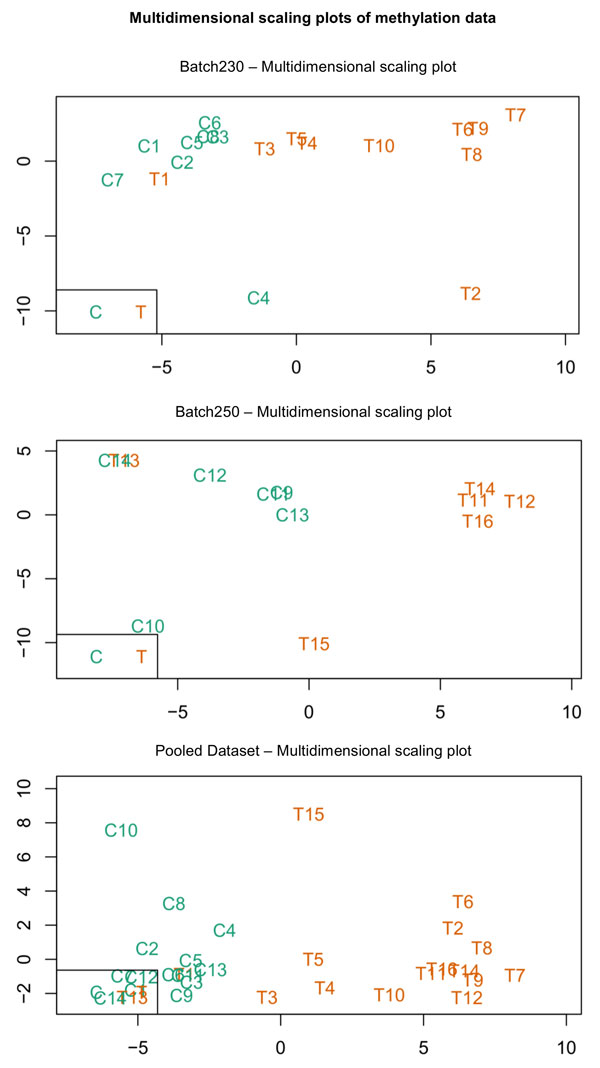
**Multidimensional scaling plots for batch230, batch250 and the pooled dataset**. Visualisation of the similarity of the normalized methylation samples based on top 1000 most variable probes among all samples in Batch230, Batch250 and the pooled dataset. Label "C" refers to Control samples (coloured in green), Label "T" refers to Tumour samples (coloured in orange). The expected was to see control and tumour samples discretely. When looked at the plots, there is one tumour sample (T1) for Batch230 and one tumour sample (T13) for Batch250 which are observed closer to the control groups. When looked at the pooled data, although two tumour samples (T13 and T1) are seen as nested with control samples, there is more discrete difference observed between Tumour and Control samples.

**Figure 2:**
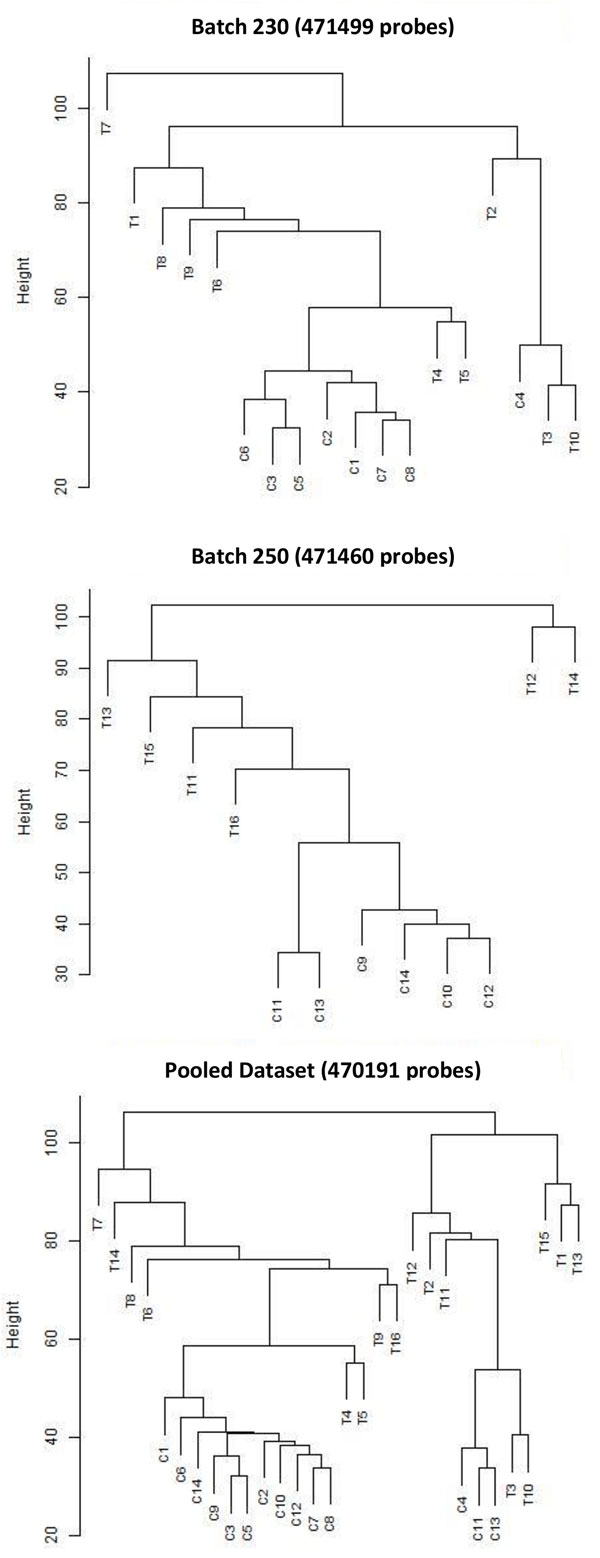
**Clustering images for each dataset in our analysis**. An alternative to Multidimensional Scaling Plots to visualise the similarity of samples based on all probes using hierarchical clustering. Label "C" refers to Control samples, Label "T" refers to Tumour samples. The expected was to see control and tumour samples discretely. When looked at the plots, control samples were discretely separated from tumour samples in the pooled dataset.

After obtaining intensity data from TCGA, intra-array normalization is done using BMIQ normalization method [40] to avoid the bias introduced by the Infinium type 2 probe design. In order to assess the similarity of normalized methylation samples in both batches and the pooled data, multidimensional scaling plots based on top 1000 most variable probes and corresponding hierarchical clustering plots are shown in Figures 1 and 2. When looked at the MDS and clustering plots, not all tumour samples were clustered together and specifically in Batch230, control samples were in separate clusters. In order to validate the problem, we have conducted the same analysis three times by double-checking the parameters. Overall, the picture was better for the pooled dataset, where there were precise "control" clusters in the plot. Adding that TCGA is a well-designed database, we had doubts on excluding the outlying samples and thus, we have continued our analysis without any elimination but focusing on pooled dataset. The reason behind enhanced performance of pooled data against individual batch data may be due to the fact that pooled data increases the confidence rate of measuring methylation and expression levels in genes, leading to an increase in the significance corresponding to each gene.

After BMIQ normalization, magnitude of batch effects are assessed and corrected using the ComBat normalization method, which is an empirical Bayes based method to correct for technical variation related to the slide [41]. After pre-processing, analysis for Copy Number Aberrations (CNA) and segmentation of methylation variable positions (MVPs) into biologically relevant differentially methylated regions (DMRs) was conducted using the "champ.MVP" function of CHAMP package. In order to have better knowledge about false positive results, Benjamini-Hochberg calculation [42] is applied for all p-values.

### RNA sequencing analysis

RNA sequencing analysis for both batches are conducted using edgeR [43] which is available as a Bioconductor [44] package. It was not possible to download raw sequencing data from TCGA Server, hence quality control, pre-processing, mapping and counting procedures were carried out by the providers of the data [6]. We have worked on counting data and applied EdgeR for detection of differential expression between tumour and control samples, which benefits from empirical Bayes estimation and tests based on the negative binomial distribution [43]. Similar to the methylation analysis, Benjamini-Hochberg correction [42] is conducted for all p-values.

### Combining significance values

For each gene, expression and methylation significances (Benjamini-Hochberg false discovery rates) are combined using survcomp package [45] which is a R [46] package that provides functions to assess and to compare the performance of risk prediction models.. In more detail, Fisher's weighted Z-method is applied while merging expression and methylation data.

$$Z_{w}=\frac{w_{X}\frac{\sqrt{n_{X}}\bar{X}}{{\hat{S}}_{X}}+w_{Y}\frac{\sqrt{n_{Y}}Ȳ}{{\hat{S}}_{Y}}}{\sqrt{w_{X}^{2}+w_{Y}^{2}}}$$

As suggested by Zaykin et al. [47] weights are assigned as square root N, where N = sample sizes. Functional enrichment results obtained from separate batches are integrated in a similar fashion (**Dataset option C for each analysis model**).

HumanMethylation450k chip informs about methylation in 450,000 different regions, whereas RNA Sequencing is not region-specific, hence it informs about genes instead of specific regions leading to discrepancy between methylation and expression. As an alternative to individual differential expression and differential methylation results we have merged the two by simply combining the significance values corresponding to each gene. However, regarding the methylation data, there was more than one differentially methylated region falling into the borders of same gene which was causing discrepancy in the data. In our analysis, we have selected the region with the most significant methylation change for each gene. Hence although there were a total of 98366 significantly altered regions for the pooled dataset, after filtering the regions which fall into the same gene, there were a total of 4530 affected unique genes at the end.

### Functional enrichment

Functional analysis for each data set is conducted via PANOGA Functional Enrichment tool [48]. PANOGA incorporates protein-protein interaction information while extracting significant pathways. It helps to identify disease related genes and devise functionally essential KEGG pathways through the identification of genes within the pathways.

PANOGA analysis for results of ten different analysis models were conducted with the help of Cytoscape [49]. At Cytoscape, we have benefitted from JactiveModules package [50] and while using JactiveModules "Number of Modules" was set as 1000 and overlap threshold was set as 0.5.

However, before giving gene lists as an input to PANOGA we have noticed that some of the genes observed in methylation results were not observed in expression results. For example, PLEC1 gene. As there was no expression information regarding PLEC1 gene, we have excluded that gene from the analysis hence there were a total of 452 genes filtered out in a similar way.

At our analysis, in order to understand the biological distribution of our genes, Gene Ontology (GO) [51] analysis is conducted using ConsensusPathDB functional annotation tool [52]. We have used the option of "over-representation analysis" and queried our gene list against Gene Ontology Level 4: Biological Process database with the p-value cut-off of 0.01. While interpreting the results of ConsensusPathDB, we have searched for 20 most important annotation clusters that were defined by DAVID functional enrichment clustering [53]. Moreover, we have conducted KEGG functional analysis for each term to understand the association between the genes inside of GO terms and the cancer state.

Particularly for our case, significant alteration at post-translational modification and regulation of transcription pathways were of higher importance as they possess the potential of affecting many biological processes. In order to find out the genes with critical effects, we have searched for transcription factors in TFCat database [54].

### Analysis performance measure

In order to evaluate different analysis strategies, we have extracted the list of thyroid cancer-related pathways and genes from previous thyroid cancer researches. For each data and significance merging strategy, our main performance measure was to observe thyroid related pathways in top 20 rankings.

On the other hand, for the purpose of understanding whether combining expression and methylation information results in better significance values for thyroid-cancer associated genes, we have compared significances of differential expression, differential methylation and combination of expression and methylation for Batch230, Batch250 and the Pooled dataset. At these tables, we have also included the information of methylation level change for all cases, which is taken as difference in Beta-value corresponding to each gene between two experiment conditions.

### Methylation change threshold analysis

With the aim of comparing the effects of putting various threshold levels for methylation change, a custom script (will be made available upon request) was written which computes inverse correlation between expression and methylation for all regions in the dataset. We have only included the regions having differential methylation and differential expression significance (FDR) below 0.1, which was picked not to be too stringent.

Simply, if methylation of a certain gene is upregulated and expression of the same gene is downregulated, that gene is counted as "inversely correlated". Same is applied for vice versa and as a next step, the ratio between number of inversely correlated genes and total number of genes are calculated for all datasets; in our case Batch230, Batch250 and the pooled dataset.

Lastly, the difference in ratio between above and below varying thresholds in the range of 0.05 (5%) and 0.5 (50%) are computed in order to find out the optimal threshold which favours inverse correlation. This way, for example if the threshold is set at 25%; number of regions with methylation change bigger than 25% and number of regions with methylation change less than 25% are compared considering the inverse correlation between expression and methylation at that certain gene. Inverse correlation gains corresponding to each dataset is linearly added together and the threshold with highest overall inverse correlation gain was picked as the best-performer.

## Results

### Methylation analysis

While exploring differentially methylated regions, we have only included the regions having a False Discovery Rate (FDR) lesser than 0.01. As a result, we had a list of 1807 significant differentially methylated regions in 1310 different genes for Batch230 and 946 differentially methylated regions in 730 different genes for Batch250. When both batches were pooled together, we were able to obtain 9333 differentially methylated regions in 4729 different genes.

### RNA sequencing analysis

Likewise, in RNA Sequencing analysis we have only included genes with False Discovery Rate (FDR) lesser than 0.01. As a result of the analysis, there were 2610 differentially expressed genes for Batch230 and there were 1482 differentially expressed genes for Batch250. When normalized expression values of Batch230 and Batch250 were pooled together (Pooled dataset), we were able to obtain 4790 differentially expressed genes. (Additional File 1).

### Methylation threshold analysis

**Figure 3:**
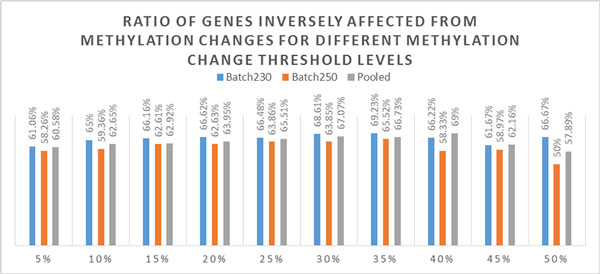
**Ratio of genes inversely affected from methylation changes for different methylation change threshold levels**. Figure showing ratio of inversely affected genomic regions to total number of affected genomic regions with differential methylation (FDR<0.01) for different methylation change threshold values. Results contain analysis for Batch230, Batch250 and pooled dataset separately. For both Batch230 and Batch250 highest ratio of inverse correlation between methylation and expression is reached with the threshold 35%. For the pooled dataset on the other hand, threshold level of 40% have highest ratio of inversely affected regions.

In order to test whether setting a valid threshold for methylation change yields enhanced functional enrichment results, an analysis is done for varying thresholds between 0.05 (5%) and 0.5 (50%) focusing on inverse correlation between expression and methylation. In both Batch230 and Batch250, genes with methylation change larger than 35% yielded highest ratio (69.23%, 66.45% respectively) of inverse correlation with expression. In the pooled dataset on the other hand, setting 40% methylation change threshold enabled us to reach highest inverse correlation ratio (69.00%) (Figure 3).

**Figure 4:**
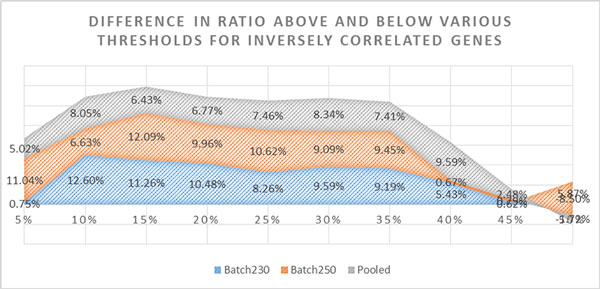
**Difference in ratio above and below various thresholds for inversely correlated genes**. Figure showing the difference in ratio of inversely correlated genomic regions (methylation ↑ expression ↓ and vice versa) above and below varying thresholds. Only regions having differential methylation FDR<0.01 are included. Results contain analysis for Batch230, Batch250 and pooled dataset separately. Considering all three datasets, best performing threshold with highest difference between above and below threshold is 15%. Hence, including threshold of 15% methylation level change in the analysis may improve the knowledge of disease aetiology.

Optimal threshold would be the one that maximizes the difference between ratios above and below of a certain threshold. Although genes having more than 40% methylation change may be informative about the disease state, setting a 40% threshold may not be beneficial for finding the optimal results. At our analysis, highest gain of inverse correlation ratio (29.77%) was obtained by using the threshold of 0.15 (15%) (Figure 4).

### Functional enrichment analysis

Ten different analysis models and their short summaries are shown in Table 1. Results regarding first four models are represented in Table 2 and next four models in Table 3. For each of these categories we have set four different result reporting options; only Batch230 results, only Batch250 results, combination of individual batch results (Pathways combined dataset) and Batch230+Batch250 (Pooled) dataset results. In the pooled dataset as threshold of 40% yielded highest ratio of inversely correlated genes, we have also made comparison between thresholds of 40% and 15% (Table 4). As a result, setting a methylation change threshold of 15% clearly outperformed setting a threshold of 40%. Moreover, we have compared the effects of inverse and positive correlation and whether which model informs more about the disease state (Table 5). When only inverse correlated genes were taken, we have observed 9 pathways in top 20 rankings (**Model 8**) and when only positively correlated genes were taken (**Model 9**), we have observed 8 pathways in top 20 rankings. On the other hand, when no filter applied and all genes above the 15% threshold were taken, we were able to reach the optimal analysis model with 12 pathways in top 20 (**Model 7**).

**Table 1:** Analysis models and their short descriptions.

Analysis Models	Model Descriptions (FDR<0.01 for all models)
**Model 1**	Only differentially expressed genes

**Model 2**	Only differentially methylated regions

**Model 3**	Differentially expressed and differentially methylated genes

**Model 4**	Significant genes when methylation and expression significances combined

**Model 5**	Genes with more than 15% methylation change and inversely correlated with expression

**Model 6**	Genes with more than 15% methylation change

**Model 7**	Significant genes with methylation level change more than 15% and obtained after combining methylation and expression significance values

**Model 8**	Significant genes with methylation level change more than 15%, inversely correlated with expression and obtained after combining methylation and expression significance values

**Model 9**	Significant genes with methylation level change more than 15%, positively correlated with expression and obtained after combining methylation and expression significance values

**Model 10**	Significant genes with methylation level change more than 40% and obtained after combining methylation and expression significance values

Short summary of each analysis model. For the search of finding the optimal analysis strategy, we have applied 10 different analysis models on different data selection options.

**Table 2:** Rankings of functional enrichment results - Part 1.

	Model 1 (Differential Expression FDR<0.01)				Model 2 (Differential Methylation FDR<0.01)				Model 3 (Both Diff. Meth and Diff. Expressed FDR<0.01)				Model 4 (Meth, Expr. Significances Combined FDR<0.01)			
**KEGG TERMs**	**A**	**B**	**C**	**D**	**A**	**B**	**C**	**D**	**A**	**B**	**C**	**D**	**A**	**B**	**C**	**D**

MAPK Signalling	**6**	**7**	**4**	**1**	**10**	**7**	**7**	**17**	**7**	-	**12**	**13**	**17**	**8**	**9**	**8**

ECM Receptor	**5**	**5**	**3**	**8**	26	-	52	**8**	**8**	-	**13**	**1**	**6**	**1**	**3**	**5**

ErbB Signalling	**13**	28	**20**	45	28	**1**	**6**	28	**14**	-	**20**	43	**20**	22	**20**	**7**

NF-KB Signalling	32	**11**	**17**	85	**11**	40	**18**	24	29	-	33	47	71	33	46	**17**

Wnt-β-Catenin Signalling	51	25	35	73	**14**	27	**16**	**13**	-	-	-	35	86	46	60	28

VEGF Signalling	46	47	47	105	42	**13**	23	85	36	-	42	-	85	84	82	56

Thyroid Cancer	30	52	34	66	55	42	43	-	**12**	**4**	**4**	65	88	65	74	69

Adherens Junction	34	**16**	**19**	24	21	**19**	**20**	**1**	34	**5**	**16**	**9**	21	**19**	**15**	**9**

p53 Signalling	**11**	**18**	**11**	**15**	68	-	77	59	**11**	-	**18**	23	34	**16**	22	30

TGF-beta Signalling	**3**	62	**12**	**5**	**18**	**6**	**12**	23	-	**1**	**5**	**5**	28	29	29	**19**

Notch Signalling	60	58	60	57	93	**18**	42	**6**	-	-	-	29	59	**9**	25	**13**

GnRH Signalling	61	27	42	26	32	24	24	40	31	-	38	26	73	41	54	68

Neurotrophin Signalling	**16**	**9**	**8**	**14**	**8**	**4**	**3**	30	**5**	-	**10**	24	**12**	**5**	**6**	**15**

Focal Adhesion	**8**	**1**	**2**	**7**	**1**	**2**	**1**	**2**	**3**	-	**3**	**2**	**7**	**2**	**2**	**3**

Transcr. Misregulation	42	**21**	29	65	**13**	-	40	-	-	-	-	37	31	23	28	34

Apoptosis	**17**	**19**	**16**	10	**12**	-	39	**20**	**15**	-	21	**16**	29	35	31	**6**

Pathways in Cancer	**1**	**2**	**1**	**3**	**2**	**3**	**2**	**16**	**1**	-	**1**	**3**	**1**	**2**	**1**	**2**

Toll-like receptor signalling pathway	**14**	57	24	32	67	-	76	60	28	-	32	-	102	85	92	43

Pentose-phosphate pathway	91	70	80	88	-	43	99	88	-	-	-	81	112	87	101	85

Rankings of previously identified thyroid-cancer associated pathways in PANOGA functional enrichment results. For each analysis strategy four different results are shown in order to understand differences between different data selection strategies; A) Batch230 Results B) Batch250 Results C) Functional Enrichment Results of different Batches combined D) Batch230+Batch250 (Pooled) results. Model 1 represents, genes with differential expression FDR<0.01, Model 2 represents, genes with differential methylation FDR <0.01, Model 3 represents, genes having both differential expression and differential methylation FDR <0.01, Model 4 represents, genes that have FDR<0.01 after significance values of methylation and expression are combined.

**Table 3:** Rankings of functional enrichment results - Part 2.

	Model 5 (>15% Methylation Change and Inversely Correlated)				Model 6 (>15% .Methylation Change)				Model 7 (>15% Methylation Change, Significances Combined)				Model 8 (>15% Methylation Change, Inverse Correlated,Significances Combined)			
**KEGG TERMs**	**A**	**B**	**C**	**D**	**A**	**B**	**C**	**D**	**A**	**B**	**C**	**D**	**A**	**B**	**C**	**D**

MAPK Signalling	-	-	-	45	**16**	**3**	**2**	**2**	29	**4**	**8**	**6**	**-**	-	**-**	59

ECM Receptor	**14**	-	28	**6**	**8**	-	35	**3**	**3**	**10**	**3**	**3**	**18**	-	34	**7**

ErbB Signalling	-	**15**	**13**	**5**	26	**6**	**7**	**7**	28	**8**	**13**	**10**	31	**3**	**4**	**4**

NF-KB Signalling	22	-	35	29	**18**	-	41	31	**20**	**-**	39	22	**5**	-	**19**	**11**

Wnt-β-Catenin Signalling	21	-	34	72	64	28	40	46	51	**-**	62	72	-	-	**-**	-

VEGF Signalling	25	24	**17**	51	44	34	36	37	50	30	33	30	34	21	**17**	60

Thyroid Cancer	28	**14**	**15**	88	32	27	22	106	39	**20**	22	45	28	**17**	**13**	65

Adherens Junction	**9**	**8**	**5**	**7**	25	24	**15**	**6**	26	**7**	**9**	**14**	**8**	**-**	24	**10**

p53 Signalling	-	-	-	26	-	-	-	80	40	**-**	50	54	-	-	**-**	25

TGF-beta Signalling	-	-	-	**12**	41	32	32	**9**	**10**	**3**	**4**	**13**	**-**	-	**-**	-

Notch Signalling	**5**	**9**	**2**	49	-	**4**	26	24	54	**18**	28	**12**	**-**	22	50	55

GnRH Signalling	-	**6**	23	21	**19**	33	21	40	-	41	78	56	**2**	-	**8**	37

Neurotrophin Signalling	**7**	**2**	**3**	**15**	**15**	**14**	**8**	**12**	**13**	**13**	**7**	**17**	**20**	-	**36**	21

Focal Adhesion	**4**	-	**16**	**1**	**3**	**17**	**4**	**1**	**1**	**6**	**2**	**2**	**7**	-	23	**2**

Transcr. Misregulation	-	-	-	40	30	-	48	77	58	**-**	68	51	-	-	**-**	65

Apoptosis	**2**	-	**7**	**8**	**20**	-	42	**20**	**6**	**-**	27	**4**	25	-	38	**1**

Pathways in Cancer	**3**	-	**8**	**3**	**1**	**1**	**1**	**8**	**2**	**1**	**1**	**1**	**3**	-	**11**	**3**

Toll-like receptor signalling pathway	-	-	-	75	-	-	-	-	-	**-**	-	**8**	-	-	-	**5**

Pentose-phosphate pathway	**18**	**12**	**10**	25	-	30	64	-	34	31	29	**18**	**4**	-	**14**	**17**

Rankings of previously identified thyroid-cancer associated pathways in PANOGA functional enrichment results - Part2. For each analysis strategy four different results are obtained in order to understand differences between different data selection strategies; A) Batch230 Results B) Batch250 Results C) Functional Enrichment Results of different Batches combined D) Batch230+Batch250 (Pooled) results. Model 5 represents, genes having more than 15% methylation change and are inversely correlated with expression values, Model 6 represents genes having more than 15% methylation change, Model 7 represents genes having more than 15% methylation change and having FDR<0.01 after significance values of methylation and expression are combined and finally Model 8 represents, genes having more than 15% methylation change, inversely correlated with expression values and having FDR<0.01 after significance values of methylation and expression are combined.

**Table 4:** Ranking comparison for 15% threshold level; between positive correlation, inverse correlation and all together.

	Model 7 (>15% Methylation Change, Significances Combined)	Model 8 (>15% Methylation Change, Inverse Correlated,Significances Combined)	Model 9 (>15% Methylation Change, Positively Correlated,Significances Combined)
**KEGG TERMs**	**Pooled Dataset**	**Pooled Dataset**	**Pooled Dataset**

MAPK Signalling	**6**	59	**7**

ECM Receptor	**3**	**7**	**4**

ErbB Signalling	**10**	**4**	**5**

NF-KB Signalling	22	**11**	-

Wnt-β-Catenin Signalling	72	-	-

VEGF Signalling	30	60	53

Thyroid Cancer	45	65	27

Adherens Junction	**14**	**10**	**11**

p53 Signalling	54	25	-

TGF-beta Signalling	**13**	-	42

Notch Signalling	**12**	55	49

GnRH Signalling	56	37	50

Neurotrophin Signalling	**17**	21	**6**

Focal Adhesion	**2**	**2**	**1**

Transcr. Misregulation	51	65	-

Apoptosis	**4**	**1**	-

Pathways in Cancer	**1**	**3**	**3**

Toll-like receptor signalling pathway	**8**	**5**	-

Pentose-phosphate pathway	**18**	**17**	**15**

Rankings of KEGG functional enrichment results on pooled dataset to investigate the differences between positive and inverse correlation. When only inverse correlated genes were taken, we observed 9 pathways in top 20 rankings and when only positively correlated genes were taken, we observed 8 pathways in top 20 rankings. On the other hand, when no filter applied and all genes above the 15% threshold were taken, we reached the optimal analysis model with 12 pathways in top 20.

**Table 5:** Ranking comparison between thresholds of 15% and 40% in the pooled dataset.

	Model 7 (>15% Methylation Change, Significances Combined)	Model 10 (>40% Methylation Change, Significances Combined)
**KEGG TERMs**	**Pooled Dataset**	**Pooled Dataset**

MAPK Signalling	**6**	-

ECM Receptor	**3**	**2**

ErbB Signalling	**10**	**10**

NF-KB Signalling	22	-

Wnt-β-Catenin Signalling	72	-

VEGF Signalling	30	24

Thyroid Cancer	45	-

Adherens Junction	**14**	**13**

p53 Signalling	54	-

TGF-beta Signalling	**13**	-

Notch Signalling	**12**	-

GnRH Signalling	56	**15**

Neurotrophin Signalling	**17**	**9**

Focal Adhesion	**2**	-

Transcr. Misregulation	51	-

Apoptosis	**4**	**1**

Pathways in Cancer	**1**	-

Toll-like receptor signalling pathway	**8**	-

Pentose-phosphate pathway	**18**	-

Rankings of KEGG functional enrichment results on pooled dataset. After combining methylation and expression significances, genes having FDR<0.01 and having methylation change >15% and >40% are compared. As a result, threshold level of 15% is better at detecting thyroid related pathways.

Overall, **Model 7 **was superior to other models at finding thyroid cancer related pathways in top 20 functional enrichment rankings. From this reason, identification of important transcription factors and more detailed functional enrichment analysis using ConsensusPathDB are conducted for the genes in Model 7 (Additional File 2 & Additional File 3).

### Thyroid cancer - associated genes

We have investigated thyroid cancer-associated genes with respect to their methylation and expression significances in our datasets (Tables 6, 7, 8). Out of 25 thyroid-cancer associated genes retrieved from previous researches, for Batch230 there were a total of 6 differentially methylated and 13 differentially expressed genes whereas for Batch250 there were only two differentially methylated and nine differentially expressed genes with FDR<0.01. On the other hand, we observed a decent increase in the numbers of thyroid cancer-associated genes for the pooled dataset where 16 of the genes were found as differentially methylated and 19 as differentially expressed.

**Table 6:** Methylation, expression analysis of Batch230 focusing only on thyroid-cancer associated genes.

	Batch230			
	
	DMR(FDR)	DE (FDR)	FDRs Combined	Methylation Change (percentage)
RAP1GAP	9.85E-04	4.42E-10	1.28E-11	**-15.75%**

TIMP3	-	-	-	**-28.98%**

DAPK	2.61E-03	2.38E-06	1.23E-07	**24.97%**

SLC5A8	-	-	1.03E-03	-3.96%

RARB	-	7.41E-03	2.67E-03	-3.22%

TSHR	2.98E-03	-	7.10E-03	-9.23%

RASSF6	2.59E-04	-	1.54E-03	**-37.42%**

CDKN2A	-	6.15E-05	1.22E-04	3.38%

MLH1	-	-	-	-1.34%

FN1	-	1.86E-09	1.27E-09	**-33.47%**

FOXE1	-	-	-	1.17%

HGF	-	-	-	-14.98%

KRT19	-	4.26E-11	1.64E-10	**-33.47%**

LGALS3	8.55E-03	1.27E-13	3.85E-14	**-11.78%**

MET	-	1.13E-17	7.35E-18	**-46.44%**

RET	-	1.22E-03	4.33E-04	**-18.49%**

KISS1R	-	2.84E-05	3.77E-05	-4.81%

ADAMTS5	-	3.53E-03	8.71E-04	**27.62%**

HOXB4	-	-	-	-3.01%

TCL1B	3.50E-03	-	-	**-35.18%**

NOTCH4	-	-	9.04E-03	-12.10%

RASSF1	-	-	-	**16.56%**

PPARG	-	3.04E-03	1.21E-03	-2.92%

ALK	-	3.27E-09	9.25E-10	-2.57%

NTRK3	-	-	-	-2.15%

Batch230 Results showing Differential Methylation (DMR), Differential Expression (DE), Combination of Differential Methylation and Differential Expression Significances (FDRs Combined) and Methylation Change in %. Only the values with Differential Expression and Differential Methylation Significances below 0.01 are shown on the table. FDR>0.01 are shown as blank. Moreover, methylation change >15% are shown as bold. Combining methylation and expression values greatly improves detecting thyroid-associated-genes as significantly altered.

**Table 7:** Methylation, expression analysis of Batch250 focusing only on thyroid-cancer associated gene.

	Batch250			
	
	DMR(FDR)	DE (FDR)	FDRs Combined	Methylation Change (percentage)
RAP1GAP	-	-	8.31E-03	**-18.04%**

TIMP3	-	-	-	**-36.24%**

DAPK	-	4.38E-06	2.60E-06	**-23.55%**

SLC5A8	-	-	-	-11.37%

RARB	-	9.34E-04	5.75E-04	**-23.60%**

TSHR	-	-	-	**-17.94%**

RASSF6	-	-	-	-14.54%

CDKN2A	-	1.58E-07	1.65E-06	14.95%

MLH1	-	-	-	**-18.74%**

FN1	-	6.59E-10	3.45E-10	**-39.00%**

FOXE1	-	-	-	2.18%

HGF	-	-	-	-11.01%

KRT19	-	9.45E-09	1.17E-08	**-27.31%**

LGALS3	-	2.58E-07	1.21E-07	-2.92%

MET	-	1.10E-09	2.25E-09	**-44.72%**

RET	-	-	-	-12.35%

KISS1R	-	1.64E-04	4.23E-04	-1.33%

ADAMTS5	-	-	-	**30.39%**

HOXB4	-	-	-	-4.06%

TCL1B	8.97E-04	-	7.54E-04	**-37.49%**

NOTCH4	-	-	-	**-22.75%**

RASSF1	5.14E-03	-	-	**20.21%**

PPARG	-	-	-	7.98%

ALK	-	1.05E-03	8.55E-04	**-19.68%**

NTRK3	-	-	-	-4.02%

Batch250 Results showing Differential Methylation (DMR), Differential Expression (DE), Combination of Differential Methylation and Differential Expression Significances (FDRs Combined) and Methylation Change in %. Only the values with Differential Expression and Differential Methylation Significances below 0.01 are shown on the table. FDR>0.01 are shown as blank. Moreover, methylation change >15% are shown as bold. Combining methylation and expression values greatly improves detecting thyroid-associated-genes as significantly altered.

**Table 8:** Methylation, expression analysis of pooled dataset focusing only on thyroid-cancer associated genes.

	Pooled Dataset			
	
	DMR(FDR)	DE (FDR)	FDRs Combined	Methylation Change (percentage)
RAP1GAP	1.29E-05	4.42E-10	1.93E-13	**-32.37%**

TIMP3	-	1.46E-03	-	**-31.69%**

DAPK	2.14E-04	1.26E-11	3.35E-12	**24.69%**

SLC5A8	-	4.80E-04	1.99E-04	-11.61%

RARB	7.43E-03	6.77E-07	1.01E-07	**-17.92%**

TSHR	6.58E-06	1.05E-07	8.96E-06	**-18.12%**

RASSF6	-	1.05E-07	2.31E-08	3.85%

CDKN2A	-	1.57E-11	4.87E-11	2.92%

MLH1	-	-	-	-1.10%

FN1	7.00E-04	3.78E-16	1.16E-17	**-39.43%**

FOXE1	-	-	-	1.00%

HGF	-	7.65E-03	-	-1.54%

KRT19	2.36E-03	2.83E-18	3.17E-19	-9.87%

LGALS3	9.21E-05	1.46E-19	7.22E-22	-9.98%

MET	1.09E-04	1.99E-26	1.50E-28	**-45.69%**

RET	3.08E-03	1.01E-04	4.96E-06	**-15.86%**

KISS1R	1.08E-03	5.98E-11	2.02E-12	-4.12%

ADAMTS5	7.80E-05	-	8.94E-03	**28.55%**

HOXB4	1.07E-03	-	7.07E-03	-3.21%

TCL1B	5.29E-03	-	1.55E-06	**-35.67%**

NOTCH4	6.85E-03	4.37E-04	4.10E-05	-13.05%

RASSF1	1.62E-03	-	5.08E-03	**19.06%**

PPARG	-	1.41E-05	7.76E-06	-2.22%

ALK	4.69E-04	3.79E-13	6.63E-15	9.44%

NTRK3	-	4.74E-03	4.15E-03	-1.44%

Pooled dataset results showing Differential Methylation (DMR), Differential Expression (DE), Combination of Differential Methylation and Differential Expression Significances (FDRs Combined) and Methylation Change in %. Only the values with Differential Expression and Differential Methylation Significances below 0.01 are shown on the table. FDR>0.01 are shown as blank. Moreover, methylation change >15% are shown as bold. Combining methylation and expression values greatly improves detecting thyroid-associated-genes as significantly altered.

When significance values of differential methylation and differential expression were combined for each gene, we were able to capture two additional genes (SLC5A8 and NOTCH4) for Batch230 and one additional gene (RAP1GAP) for Batch250. Upon performing the same analysis for the pooled dataset, we observed 18 differentially altered genes, which was the highest compared to the previous dataset options. The results for the pooled dataset covered all of the genes that were captured on individual batch results, therefore besides combining significance values, pooling, i.e. expanding the dataset, aids at capturing disease-related genes with higher ratio.

## Discussion

For the purpose of understanding the interplay between expression and methylation in thyroid cancer, we have conducted comparisons between four data and ten analysis strategies with respect to the observance rate of thyroid related pathways in the functional enrichment results (Tables 2, 3, 4, 5). Moreover, we have also conducted a threshold analysis to understand whether setting a methylation change threshold improves the outcome of the experiment.

### Methylation threshold analysis

In order to identify the benefits of setting a methylation level threshold, we have conducted a threshold analysis for various threshold levels by calculating the inverse correlation ratio between methylation and expression. When only inverse correlation ratios above different thresholds were looked at, best performing threshold was 35% for both Batch230 and Batch250 and 40% for the pooled dataset (Figure 3). However, the reason behind setting a threshold is to witness a concrete difference between above and below thresholds. In this sense, optimal threshold would be the one that maximizes the difference between ratio above and below of a certain threshold.

When investigating the total inverse correlation gain for all three datasets, best performing threshold level was found at "15%" with 29.77% correlation gain where improvement in inverse correlation between change in methylation level and expression reached its highest value (Figure 4).

Consequently, when 15% methylation change threshold was added to **Model 4**, which previously possessed maximum number of thyroid-cancer associated pathways in top20 functional enrichment rankings, we were able to reach the optimal analysis strategy with 12 thyroid-cancer associated pathways in top 20 rankings (**Model 7**) (2, 3, 4, 5). Similarly, when **Model 2 **and **Model 6 **were compared to each other, addition of 15% methylation change threshold improved the functional enrichment results by additionally identifying ErbB signalling, TGF-beta signalling and Neurotrophin signalling pathways in top 20 rankings. Thus, it can be argued that the genes with more than 15% methylation change may be the core reason behind changes in these pathways, which were all associated with thyroid-cancer in previous works.

Moreover, we have also compared functional enrichment results between **Model 7**, 15% methylation threshold and **Model 10**, 40% methylation threshold, which did not have the highest correlation gain but had the highest inverse correlation percentage in the pooled dataset. As a result, setting 15% threshold level clearly outperformed threshold of 40% (Table 4), implying that the information of a "gain of inverse correlation" above and below the threshold is more important than "overall inverse correlation" ratio above the threshold.

### Combining methylation and expression data

Due to the reason that methylation and gene expression have different roles in the development of thyroid cancer, combining significance values obtained from methylation and expression studies leads to a better detection of thyroid-related genes (Tables 6, 7, 8). To exemplify, for Batch230, SLC5A8 gene was not detected as significantly expressed or significantly methylated. However when the significances of expression and methylation were combined, we observed SLC5A8 as significantly altered with false discovery rate of 0.001. Similar cases were also observed for Batch250 and pooled dataset, hence combining methylation and gene expression information on pooled data enabled us to obtain highest ratio (21 out of 25) of detecting thyroid-cancer associated genes as significantly altered.

Moreover, for the purpose of understanding the reflection of combining methylation and expression significances on functional enrichment results, we have compared **Model 6 **(>15% methylation change) with **Model 7 **(>15% methylation change and methylation, expression significances combined) and **Model 4 **(Only methylation, expression significances combined) with **Model 1 **(Only differential expression) and **Model 2 **(Only differential methylation).

Considering the pooled dataset, for **Model 6**, we have observed 9 thyroid-cancer associated pathways in top20 functional enrichment results whereas for **Model 7**, which is the same dataset with only methylation and expression significances were combined, we have detected 12 thyroid-cancer associated pathways in top20 functional enrichment results. Similarly, for **Model 1 **there were 7 and for **Model 2 **there were 8 thyroid-cancer associated pathways in top20 functional enrichment results. When expression and methylation significances were combined instead of treated separately, we were able to observe 11 important pathways in top 20 functional enrichment results (**Model 4**). Moreover, there were various pathways that were not captured at all in **Model 1 and 2**, which were only captured when the significances of expression and methylation were combined. For example; in Batch250 differential methylation functional enrichment results (**Model 2**, B dataset), p53 signalling pathway was not listed as significant at all, with Bonferroni Score above 0.01. When methylation and expression significances were combined (**Model 4**, B dataset), p53 signalling pathway was observed at 16th rank with Bonferroni Score 1.51E-12. Similar improvement was also observed among **Model 5 **and **Model 8**, as combining significances led to an improved performance with additional detection of toll-like receptor pathway in top 10 rankings.

Consequently, incorporating methylation and expression information together not only improved detection rate of disease-specific genes but it also increased the rankings of disease-specific pathways in functional enrichment results.

Overall, when the data was pooled, methylation, expression significances were combined and only genes with more than 15% methylation change were selected, best performing results were reached with 12 pathways in top20 functional enrichment results (Table 2, 3, 4, 5) namely; MAPK signalling, Extracellular matrix receptor, ErbB signalling, TGF-beta signalling, Notch signalling, Neurotrophin signalling, Apoptosis, Focal adhesion, Pathways in cancer, Toll-like receptor signalling, Pentose-phospate and Adherens junction pathways.

### Testing on an independent dataset

In addition to the supporting articles from the literature, for the purpose of proving the generalizability and efficiency of our proposed framework, we have applied the same procedures described above on another independent dataset with 30 samples retrieved from thyroid cancer experiments in TCGA. To achieve that, firstly we have calculated the methylation threshold value with "maximum inverse correlation gain", which was also 15% for the test dataset and secondly, we have combined methylation and expression significances by using Fisher's weighted Z-method. As a result, compared to our training dataset results, we were able to obtain similar pathways in similar rankings in the test dataset, hence there were 11 thyroid cancer-associated pathways in top 20 functional enrichment rankings (Table 9). These findings also support that our approach can be applied to different, independent cancer datasets, which may aid at detecting important pathways for other cancer types as well.

**Table 9:** Validating the proposed framework with a new dataset.

	Training Dataset (>15% Methylation Change, Significances Combined)	Test Dataset (>15% Methylation Change, Significances Combined)
**KEGG TERMs**	**Pooled**	**Pooled**

MAPK Signalling	**6**	**11**

ECM Receptor	**3**	**1**

ErbB Signalling	**10**	**16**

NF-KB Signalling	22	25

Wnt-β-Catenin Signalling	72	80

VEGF Signalling	30	69

Thyroid Cancer	45	68

Adherens Junction	**14**	**19**

p53 Signalling	54	**17**

TGF-beta Signalling	**13**	**7**

Notch Signalling	**12**	83

GnRH Signalling	56	**15**

Neurotrophin Signalling	**17**	**12**

Focal Adhesion	**2**	**2**

Transcr. Misregulation	51	-

Apoptosis	**4**	**20**

Pathways in Cancer	**1**	**4**

Toll-like receptor signalling pathway	**8**	-

Pentose-phosphate pathway	**18**	75

Comparison between the training and test dataset. For the training dataset, optimal results were obtained using **Model 7**. When the same analysis procedure of training dataset is applied to the test dataset (30 samples), similar results were obtained. There were 12 KEGG functional annotation terms for the training dataset whereas this number was 11 for the test dataset, which shows that our proposed framework is also applicable to independent datasets.

### Disease aetiology

Although there may be other mechanisms at play leading to the thyroid cancer state, in this work we have mainly investigated pathways which were mainly influenced by expression changes highly correlated with methylation changes. While searching for the optimal model, several common pathways were observed at different rankings in almost all of the models, reassuring that methylation change may disturb certain pathways that might be involved in thyroid cancer aetiology. When focusing only on differential methylation results, we have observed significant changes in important pathways such as MAPK Signalling, Wnt-β-catenin Signalling, Notch signalling, Apoptosis and TGF-beta signalling pathways. Besides the pathways that were directly affected by methylation, other secondary molecular mechanisms were also triggered, such as Transcriptional misregulation, Thyroid cancer and p53 signalling pathways, which were only captured by expression experiments. Specifically when pooled data results which possess more than 40% methylation change were being investigated (Additional File 4), we observed significant changes in Apoptosis, Extracellular matrix, ErbB, VEGF, GnRH and Neurotrophin signalling pathways. Thus, it is more probable that the core reason behind major changes in these pathways may be due to high methylation level change between disease and normal state (Table 4).

In our analysis, optimal analysis strategy which yielded maximum number of thyroid-cancer associated pathways in top rankings was found to be **Model 7**. When the functional enrichment results of the best-performing analysis model was investigated in detail, all of the top20 ranked pathways on the list could be associated with thyroid cancer (Additional File 2). In addition to the thyroid-cancer related pathways that were extracted from literature at the beginning, Endocytosis [55], Glutamate [56], Proteasome [57], Gluconeogenesis and glycolysis [58] pathways are found as linked to thyroid cancer in previous works about thyroid cancer.

Furthermore, when the details of 2826 genes that have >15% methylation change were explored, some of the GO: Biological Process terms with high significance were: regulation of signal transduction, cell differentiation, phosphate containing metabolic process, morphogenesis and neuron development. For each annotation term, we have performed KEGG functional analysis to examine the association with the cancer state (Table 10). Almost all of the terms were found to be associated with "Pathways in Cancer" which was also supported by the recent literature works [16,37,59-66].

**Table 10:** GO - Biological process functional annotation results for Model 7.

GO: Biological Process Terms	No. of genes that overlap with our list	Associated q-value	No. of genes in cancer pathway	Association with cancer pathway
Regulation of Signal Transduction	540 (21.5%)	1.54E-29	56	2.06E-15

Cellular Development Process, Cell Differentiation	633 (19.2%)	2.16E-21	74	4.02E-23

Phosphate Containing Compound Metabolic Process	653 (19.1%)	2.16E-21	59	2.35E-14

Anatomical Structure Formation, Morphogenesis	271 (22.7%)	8.04E-17	34	1.72E-11

Neuron Development	229 (23.9%)	8.47E-17	29	1.57E-09

Actin Cytoskeleton Organization	142 (27.6%)	1.53E-15	13	1.46E-03

Regulation of Catalytic Activity	411 (19.8%)	3.30E-15	48	5.03E-15

Circulatory System Development	199 (23.3%)	1.67E-13	40	1.45E-19

Cell Junction Assembly	70 (33.8%)	3.87E-12	10	3.12E-04

Vasculature Development	139 (24.9%)	1.28E-11	28	1.27E-13

Regulation of Adhesion	137 (24.7%)	2.71E-11	25	1.46E-11

Regulation of Programmed Cell Death	339 (19.3%)	5.69E-11	48	2.25E-17

Protein Kinase Activity	180 (22.0%)	4.18E-10	30	3.16E-15

Response to External Stimulus	142 (23.1%)	1.83E-09	20	4.95E-07

Epithelium Development	214 (20.5%)	4.47E-09	46	1.07E-25

Response to Growth Factor	151 (22.1%)	9.85E-09	35	5.21E-19

Protein Modification Process	526 (17.0%)	5.44E-08	57	6.96E-17

Regulation of Developmental Process	190 (19.6%)	1.19E-06	36	7.49E-17

Regulation of Cell Growth	71 (21.5%)	4.62E-05	9	3.88E-03

Mesonephros Development	27 (26.0%)	3.82E-04	12	2.08E-10

Biological Process annotation table for significantly altered genes in **Model 7 **obtained using ConsensusPathDB. Out of 340 GO: Biological Process terms with q-value <0.01, information of 20 important terms are reported. For each annotation term in the list, we have conducted KEGG Pathway Analysis. Almost all of the terms were significantly associated with "Pathways in Cancer".

Moreover, since post-translational modification and regulation of transcription pathways are critical for cancer diagnosis and therapy [67,68], we have searched for transcription factors in TFCat database [54] and as a result, 207 out of 2826 genes (7.32%) were annotated as transcription factors (**S2 Table**) and 245 out of 2826 genes (8.66%) were annotated as being involved in post-translational modification processes with Benjamini significance of "7.98E-05" in ConsensusPathDB analysis. Consequently, these genes may be active at altering other pathways, revealing other mechanisms involved in thyroid cancer.

## Conclusion

Overall, we define a comprehensive analysis strategy for incorporating methylation and expression information, which enables detection of primary and secondary mechanisms associated with the thyroid cancer. As a result of our case study, incorporating methylation and expression information is a viable strategy at detecting disease-related genes and disease-related pathways more efficiently. Moreover, while increasing the number of samples improves the analysis confidence of the experiment, optimal results with respect to disease-related pathways were obtained after setting a valid threshold for change in methylation level, which is defined by considering the inverse correlation gain above and below of a certain threshold. From biological perspective, MAPK signalling, Extracellular matrix, Focal adhesion, ErbB signalling, Apoptosis, TGF-beta signalling, Glutamatergic synapse and Toll-like receptor signalling pathways were found as significantly altered in our analysis, hence these pathways may be the core pathways that are involved in thyroid cancer. Furthermore, significantly altered transcription factors and post-translational modifiers distinguished by our analysis strategy may be crucial at identifying secondary mechanisms lying behind thyroid cancer. We believe that our approach on incorporating methylation and expression data reveals insights of thyroid cancer which cannot be extracted using only methylation or only expression data.

## Competing interests

The authors declare that they have no competing interests.

## Authors' contributions

BO and US wrote the article together. US was the advisor in the whole procedure. BO performed all analyses including DNA methylation and RNA-Seq expression analysis. Both authors have read and approved the manuscript for publication.

## Supplementary Material

Additional file 1 **Gene Expression MA plots of Batch230, Batch250 and Pooled Dataset**. Vertical axis represent log ratios between two measurements, which are colored in black and red. Horizontal axis represent mean values of two measurements. Click here for file

Additional file 2 Panoga Top20 functional enrichment results when methylation and expression significances are combined for the Pooled dataset, and only genes with >15% methylation change are selected (Model 7). Click here for file

Additional file 3 **List of transcription factors that have more than 15% methylation change in pooled dataset**. Click here for file

Additional file 4 **Top 20 functional enrichment result for the pooled dataset with genes having >40% methylation change**. Click here for file

## Declarations

Publication charges for this article have been funded by Acibadem University.

This article has been published as part of *BMC Genomics *Volume 16 Supplement 12, 2015: Joint 26th Genome Informatics Workshop and 14th International Conference on Bioinformatics: Genomics. The full contents of the supplement are available online at http://www.biomedcentral.com/bmcgenomics/supplements/16/S12.
