# Supplementary material for: A novel analysis strategy for integrating methylation and expression data reveals core pathways for thyroid cancer aetiology
Source: BMC Genomics. 2015 Dec 9;16(Suppl 12):S7. doi: 10.1186/1471-2164-16-S12-S7 (PMC4682414; doi:10.1186/1471-2164-16-S12-S7)

Additional File 1. Gene Expression MA plots of Batch230, Batch250 and Pooled Dataset.

Vertical axis represent log ratios between two measurements, which are colored in black and red. Horizontal axis represent mean values of two measurements.

MA plot of Batch230

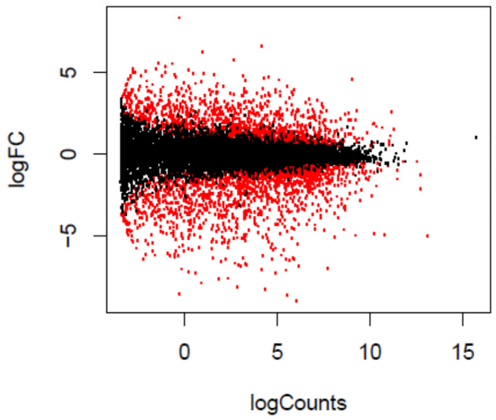

MA plot of Batch250

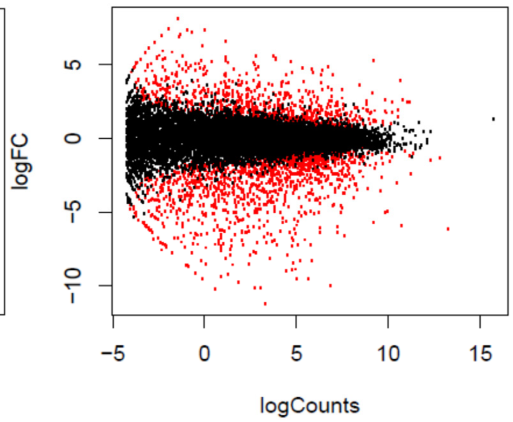

MA plot of Pooled Dataset

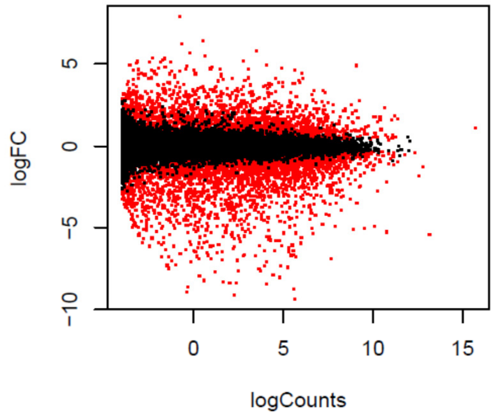

Supplement: Additional file 1 — Gene Expression MA plots of Batch230, Batch250 and Pooled Dataset. Vertical axis represent log ratios between two measurements, which are colored in black and red. Horizontal axis represent mean values of two measurements. [file 1471-2164-16-S12-S7-S1.pdf]
