# Supplementary material for: A novel analysis strategy for integrating methylation and expression data reveals core pathways for thyroid cancer aetiology
Source: BMC Genomics. 2015 Dec 9;16(Suppl 12):S7. doi: 10.1186/1471-2164-16-S12-S7 (PMC4682414; doi:10.1186/1471-2164-16-S12-S7)
Supplement: Additional file 2 — Panoga Top20 functional enrichment results when methylation and expression significances are combined for the Pooled dataset, and only genes with >15% methylation change are selected (Model 7). [file 1471-2164-16-S12-S7-S2.pdf]

Table S1

KEGG Term	Bonferroni P-values	Times Found	Pathway Associated Genes Found in Subnetworks
Pathways in cancer	2,35E-26	12	ITGB1, RET, CDKN1A, CDKN1B, FGF1, ETS1, GLI3, IGF1R, CCND1, MYC, SUFU, AKT1, EP300, HRAS, JAK1, PDGFRA, MAP2K1, ITGA2, AXIN1, PRKCA, AXIN2, FOS, RUNX1, MSH6, TRAF6, RARA, RARB, MET, CTBP2, CTBP1, EPAS1, MAX, PIK3R1, EGFR, RXRB, RXRA, DVL1, RXRG, STAT5A, STAT5B, CREBBP, TCF7L1, STAT1, STAT3, PTK2, BCL2, CTNNB1
Focal adhesion	1,29E-23	20	ITGB1, RASGRF1, TNC, ACTB, IGF1R, MYLK, CCND1, CAPN2, RAC2, AKT1, RAC1, HRAS, PRKCG, PDGFRA, MAP2K1, ACTN1, HGF, ITGA2, PRKCA, PIK3CA, COL4A1, ITGA6, CRK, MET, VCL, SHC3, TNXB, SHC1, SRC, LAMA3, PIK3R1, CDC42, VTN, PAK1, PAK7, FLNA, MAPK1, FYN, MAPK3, LAMB3, CAV2, EGF, FN1, BRAF, IGF1, PTK2, VEGFA, COL1A1, BCL2, CTNNB1
ECM-receptor interaction	7,66E-21	12	ITGB1, LAMA5, LAMB3, ITGA3, LAMA1, ITGA2, ITGB3, SDC2, FN1, TNC, LAMC2, LAMC1, GP5, HSPG2, THBS1, COL1A1, COL1A2, COL4A2, COL4A1, ITGA8, SDC1, ITGA6, ITGA5, CD44
Apoptosis	3,66E-20	13	TNFRSF10B, FASLG, TNFRSF10A, CFLAR, IL1RAP, PIK3R1, TNFRSF10D, TNFRSF1A, PPP3CA, CASP8, IRAK1, IRAK2, BCL2, FAS, FADD, BID, BCL2L1
Cell cycle	2,02E-18	4	CDKN1A, CDKN1B, PCNA, TGFB1, CDKN2A, CDC7, CDC6, CDC25C, PKMYT1, CDC25B, CCNA2, ORC5, CDK6, CCND1, CDK2, CDK1, MCM3, MCM5, SFN, BUB1
MAPK signaling pathway	2,45E-18	5	ATF2, RASGRF1, FGF1, PPP3CA, PPP3CC, RPS6KA2, STMN1, RAC2, AKT1, MAP3K8, RAC1, HRAS, MAP3K4, MAP3K5, PRKCG, DUSP5, PDGFRA, MAP2K1, DUSP1, PRKCA, FOS, DUSP6, CDC25B, TNFRSF1A, CACNB1, MAPKSP1, PPM1B, MAPKAPK3, TRAF6, MAPT, CRK, CDC42, PAK1, PPP3R1, RRAS, FLNA, MAPK1, CD14, MAP4K4, MAPK3, TGFB2, MAP3K1, TGFB1, EGF, NFATC2, BRAF, MAPK14, MAPK13, NR4A1, TAB1, FGFR2
Glutamatergic synapse	4,42E-16	1	GRIA1, PRKCG, HOMER2, TRPC1, ITPR1, ITPR2, ITPR3, PRKCA, CACNA1C, GRM1, DLG4, GNAQ, GRIA4
Toll-like receptor signaling pathway	4,76E-16	2	STAT1, IRAK4, PIK3R1, CASP8, TOLLIP, TRAF6, TLR8, CD14, FADD, TLR6, TLR5, MYD88, TLR3, TLR2
Endocytosis	9,62E-16	3	TSG101, SRC, CLTC, AP2A1, ASAP2, EGFR, IGF1R, CDC42, RAB11FIP1, LDLRAP1, EPS15, HRAS, LDLR, GIT1, SH3GL1, RAB4A, PDCD6IP, VPS37B, EPN2, RUFY1, RABEP1, HGS, TRAF6, MET, RAB5A, VPS28
ErbB signaling pathway	1,88E-15	36	CAMK2D, SHC3, SHC1, SRC, CAMK2A, PIK3R1, EGFR, PAK1, ERBB3, NCK2, STAT5A, STAT5B, EGF, PRKCA, PTK2
Circadian rhythm	4,13E-15	4	PER2, PER1, PER3, CSNK1D, CSNK1E, CLOCK, ARNTL
Notch signaling pathway	2,09E-14	5	JAG2, NOTCH2, JAG1, NOTCH1, MAML2, PSEN2, PSEN1, RBPJ, DLL1, DLL4, SNW1, NUMB, MAML3
TGF-beta signaling pathway	3,74E-14	5	TGFB2, TGFB1, INHBB, BMP7, ACVR2A, BMP5, BMP4, BMP2, BMPR1B, BMPR1A
Adherens junction	9,56E-14	24	SMAD2, SMAD4, TCF7L1, SMAD3, SRC, ACTN1, IQGAP1, SORBS1, BAIAP2, MLLT4, ACTB, EGFR, PTPRF, TGFBR1, CDC42, PARD3, RAC2, SNAI1, CTNNB1, MAPK1, FYN, PVRL4, MET

Long-term depression	1,19E-13	20	GRIA1, LYN, PRKCG, RYR1, ITPR1, ITPR2, ITPR3, PRKCA, GRM1, GNAQ
Glycolysis / Gluconeogenesis	4,39E-13	2	GPI, G6PC, ALDOA, FBP1, GAPDH, PFKM, FBP2, HK2, PFKP, HK1
Neurotrophin signaling pathway	6,29E-13	6	CAMK2B, SHC3, SHC1, YWHAB, CAMK2A, PIK3R1, PSEN1, CDC42, IRAK2, RPS6KA2, ABL1, AKT1, MAPK1, RAC1, HRAS, YWHAG, MAP3K5, MAPK3, MAP2K1, MAP3K1, PRKCD, BRAF, PTPN11, MAPK14, MAPK13, PIK3CA, TRAF6, BCL2, NFKBIE, CRK
Pentose phosphate pathway	8,42E-13	4	GPI, G6PD, ALDOA, TKT, FBP1, PFKM, FBP2, PFKP
Axon guidance	9,91E-13	10	ITGB1, CXCR4, SEMA3F, CDC42, PPP3CA, ABLIM1, PAK1, PPP3R1, PPP3CC, ABL1, NCK2, PAK7, RAC2, MAPK1, FYN, RAC1, EPHB2, SRGAP2, HRAS, SRGAP1, EPHB3, MAPK3, EPHA4, LIMK1, SEMA4C, NFATC2, L1CAM, PTK2, MET, NGEF
Proteasome	1,32E-12	2	PSMB8, PSMD8, PSMB7, PSMD6, PSMA1, PSMC3, PSMC4, PSMC1, PSMD2, PSME3, PSMC2, PSME1, PSMF1

Top20 PANOGA Functional Enrichment Results for pooled dataset with genes having >15% methylation change. “Times Found” refers to number of genes included at our dataset which plays role at that specific pathway. Most of the pathways observed at functional enrichment results of genes having >15% methylation change, are detected as associated with thyroid cancer in the literature.
