# Supplementary material for: A novel analysis strategy for integrating methylation and expression data reveals core pathways for thyroid cancer aetiology
Source: BMC Genomics. 2015 Dec 9;16(Suppl 12):S7. doi: 10.1186/1471-2164-16-S12-S7 (PMC4682414; doi:10.1186/1471-2164-16-S12-S7)
Supplement: Additional file 3 — List of transcription factors that have more than 15% methylation change in pooled dataset. [file 1471-2164-16-S12-S7-S3.pdf]

Table S2

Transcription Factor	Methylation FDR	Transcription Factor	Methylation FDR	Transcription Factor	Methylation FDR
FHL2	2.46E-09	MEF2D	2.21E-04	RUNX1	2.43E-03
FOXP1	6.99E-09	ETS2	2.26E-04	RORA	2.44E-03
FLI1	9.75E-09	NFIL3	2.42E-04	HDAC9	2.50E-03
RXRB	2.82E-08	HIVEP1	2.64E-04	ETS1	2.55E-03
BCL6	5.86E-08	RUNX1	2.79E-04	ESRRB	2.56E-03
HMGB2	2.47E-07	ZBTB16	2.92E-04	TBR1	2.70E-03
MEF2A	2.77E-07	GATA4	3.26E-04	STAT5A	2.92E-03
RORA	4.13E-07	STAT5B	3.31E-04	ESR2	2.97E-03
MAFF	5.22E-07	HIC1	3.48E-04	ZBTB16	3.13E-03
THRA	6.97E-07	RORA	3.64E-04	ATF3	3.16E-03
MEF2D	7.39E-07	MTA1	3.80E-04	ELK3	3.21E-03
ELF1	8.93E-07	MNT	4.13E-04	IRF9	3.28E-03
ARNTL	1.02E-06	MEOX1	4.45E-04	ZIC1	3.32E-03
MTA2	1.10E-06	NFIC	4.60E-04	HIC1	3.34E-03
CTBP2	1.17E-06	HIVEP2	4.94E-04	CBFA2T3	3.35E-03
TEAD4	1.22E-06	BCL6	5.42E-04	MLLT1	3.37E-03
KLF7	1.28E-06	TBX21	5.51E-04	ETS1	3.37E-03
MEF2D	1.67E-06	STAT5A	5.58E-04	RORA	3.40E-03
HIVEP2	2.38E-06	SREBF1	5.64E-04	ASCC1	3.42E-03
NR6A1	2.47E-06	EHF	5.91E-04	ARNT2	3.47E-03
HOXA2	3.10E-06	RARA	6.15E-04	NR2F1	3.63E-03
THRB	3.54E-06	LPP	6.69E-04	HDAC4	3.66E-03
RARG	4.91E-06	BACH2	6.74E-04	ARNTL	3.66E-03
ZBTB7B	5.33E-06	PRRX2	7.14E-04	NR2F1	3.85E-03
NFATC2	5.36E-06	STAT5A	7.16E-04	HOXA1	4.02E-03
HDAC4	5.51E-06	MAFF	7.27E-04	CUX1	4.16E-03
EHF	6.06E-06	NFE2L2	7.34E-04	CDKN1A	4.34E-03
RXRB	6.33E-06	GMEB2	7.49E-04	HOXD10	4.38E-03
BHLHE40	7.20E-06	BHLHE40	7.78E-04	CBFA2T3	4.58E-03
CDKN1A	7.58E-06	TCF7L1	8.32E-04	FHL2	4.60E-03
RARG	8.01E-06	GLI3	8.35E-04	NRG1	4.65E-03
GLI3	8.13E-06	ARID1A	8.76E-04	SP1	4.67E-03
BACH2	8.48E-06	ATF4	9.04E-04	RUNX1	5.00E-03
FOXO1	8.49E-06	NR5A2	9.07E-04	KLF4	5.11E-03
MAFF	9.75E-06	RORA	9.30E-04	BCL3	5.23E-03
POU2AF1	1.21E-05	NFIX	1.03E-03	HOXC4	5.31E-03
FOS	1.28E-05	MEF2D	1.09E-03	STAT1	5.37E-03
TEAD1	1.57E-05	GTF2B	1.09E-03	BHLHE41	5.38E-03
RUNX2	1.66E-05	ARNT2	1.10E-03	HOXA9	5.54E-03
HOXD10	1.72E-05	ELK3	1.11E-03	MEIS1	5.73E-03
PBX1	2.02E-05	CUX1	1.15E-03	KLF5	5.98E-03
EPAS1	2.07E-05	CREM	1.17E-03	FOXP2	6.00E-03
MECOM	2.07E-05	KCNIP3	1.24E-03	TRRAP	6.12E-03
KLF16	2.26E-05	NFIX	1.25E-03	SOX17	6.12E-03

TEAD1	2.65E-05	FOXP1	1.26E-03	CREB3L1	6.15E-03
HDAC5	2.70E-05	SNAI1	1.28E-03	VSX1	6.53E-03
KLF16	3.16E-05	NFE2L2	1.35E-03	RXRB	6.76E-03
HDAC4	3.80E-05	CREB3L1	1.37E-03	RXRA	6.83E-03
CREB3L1	3.91E-05	RUNX1	1.53E-03	NFE2L3	7.11E-03
HDAC9	4.37E-05	PPARGC1B	1.59E-03	HDAC9	7.14E-03
NFE2L3	5.05E-05	BHLHE40	1.61E-03	MKL2	7.21E-03
NR2F6	5.43E-05	TCF7L1	1.65E-03	RARB	7.43E-03
ELK3	5.66E-05	HDAC9	1.67E-03	RREB1	7.54E-03
BHLHE40	5.80E-05	RUNX2	1.75E-03	TBX5	7.57E-03
HDAC4	5.83E-05	RARA	1.86E-03	MEIS1	7.61E-03
PRDM1	5.85E-05	ZEB2	1.91E-03	GTF2IRD1	7.74E-03
GTF2IRD1	5.99E-05	GLI1	1.98E-03	ARID1B	8.82E-03
RORA	7.19E-05	BCL6	2.01E-03	GTF2IRD1	8.89E-03
GTF3C1	7.87E-05	KLF12	2.03E-03	TEAD1	8.90E-03
BHLHE40	7.87E-05	BCL6	2.05E-03	ZIC1	8.99E-03
RUNX1	8.26E-05	HDAC7	2.14E-03	CDKN1A	9.17E-03
KDM5B	8.38E-05	HNF1B	2.15E-03	NR1H2	9.21E-03
HDAC9	8.70E-05	EHF	2.19E-03	RARA	9.28E-03
ARNTL	9.00E-05	TCEA2	2.21E-03	MLLT1	9.31E-03
BHLHE40	9.04E-05	JDP2	2.22E-03	PBX1	9.43E-03
MAX	9.10E-05	RUNX2	2.27E-03	NFIX	9.63E-03
ETS1	1.27E-04	VSX1	2.31E-03	THRA	9.70E-03
NFIX	1.42E-04	GLI3	2.32E-03	FOXP1	9.91E-03
RREB1	1.47E-04	RUNX2	2.41E-03	CBFA2T3	9.96E-03

List of transcription factors that have methylation level change >15% and significantly methylated in pooled dataset with FDR<0.01. These genes may be crucial for disease aetiology as they may be altering other pathways by affecting various number of genes.
