# Supplementary material for: A novel analysis strategy for integrating methylation and expression data reveals core pathways for thyroid cancer aetiology
Source: BMC Genomics. 2015 Dec 9;16(Suppl 12):S7. doi: 10.1186/1471-2164-16-S12-S7 (PMC4682414; doi:10.1186/1471-2164-16-S12-S7)
Supplement: Additional file 4 — Top 20 functional enrichment result for the pooled dataset with genes having >40% methylation change. [file 1471-2164-16-S12-S7-S4.pdf]

Table S3

KEGG Term	Term Pvalues Corr Bonf	Times Found	Pathway Associated Genes Found in Subnetworks
Apoptosis	1,71E-13	1	PPP3CA*, CASP8*, CAPN2*, FAS*, RIPK1*, TRAF2*, CAPN1*, CFLAR*, FADD*, TNFRSF1A*,
ECM-receptor interaction	2,20E-12	1	ITGB1*, COL1A2*, LAMB3*, ITGA3*, ITGA2*, SDC2*, FN1*, ITGB7*, THBS1*, CD44*,
Citrate cycle (TCA cycle)	4,44E-10	2	FH*, PC*, MDH2*, SDHB*,
Melanoma	7,71E-09	2	PDGFRB*, CDK6*, MAPK1*, PDGFA*, FGF2*, MET*, EGFR*, IGF1R*, MAPK3*,
Circadian rhythm	8,09E-09	5	PER1*, PER3*, CSNK1D*, RORA*, CSNK1E*, ARNTL*,
T cell receptor signaling pathway	1,37E-07	1	CSF2*, FOS*, PPP3CA*, PPP3R1*, PPP3CC*, PTPRC*, PIK3CA*, MAPK1*, FYN*, GRB2*, SOS1*, PAK2*, MAPK3*,
Glioma	8,29E-07	2	CDK6*, CCND1*, PIK3CA*, MAPK1*, CALM3*, GRB2*, SOS1*, EGFR*, IGF1R*, MAPK3*,
Dorso-ventral axis formation	9,90E-07	2	NOTCH2*, MAPK1*, GRB2*, SOS1*, EGFR*, ETV6*, MAPK3*,
Neurotrophin signaling pathway	1,01E-06	1	NTRK1*, MAP3K3*, MAP3K1*, PRKCD*, PSEN2*, PIK3CA*, ABL1*, MAPK1*, CALM3*, GRB2*, SOS1*, MAP3K5*, MAPK3*,
ErbB signaling pathway	1,35E-06	1	CDKN1B*, PIK3CA*, SRC*, ABL1*, MAPK1*, GRB2*, SOS1*, PAK2*, EGFR*, PTK2*, MAPK3*,
Endometrial cancer	1,45E-06	1	CCND1*, PIK3CA*, CDH1*, PDPK1*, MAPK1*, GRB2*, SOS1*, EGFR*, MAPK3*,
Non-small cell lung cancer	2,05E-06	1	CDK6*, CCND1*, PIK3CA*, PDPK1*, MAPK1*, GRB2*, SOS1*, EGFR*, MAPK3*,
Adherens junction	2,61E-06	1	TJP1*, PTPN1*, CDH1*, SRC*, MAPK1*, FYN*, MET*, EGFR*, IGF1R*, MAPK3*,
Renal cell carcinoma	3,02E-06	2	EPAS1*, EP300*, MAPK1*, PTPN11*, CRK*, MET*, MAPK3*,
GnRH signaling pathway	6,43E-06	1	MAP3K3*, MAP3K1*, SRC*, PRKCD*, MAPK1*, CALM3*, GRB2*, CACNA1C*, SOS1*, EGFR*, MAPK3*,
Prostate cancer	1,75E-05	1	CDKN1B*, CCND1*, PIK3CA*, PDPK1*, MAPK1*, GRB2*, SOS1*, EGFR*, IGF1R*, MAPK3*,
Chronic myeloid leukemia	2,96E-05	1	CDK6*, CDKN1B*, CCND1*, PIK3CA*, ABL1*, MAPK1*, GRB2*, SOS1*, MAPK3*,
Bladder cancer	4,57E-05	1	CDK4*, MYC*, MDM2*, TP53*, EGFR*,
B cell receptor signaling pathway	4,69E-05	1	PPP3CA*, PPP3R1*, PPP3CC*, PIK3CA*, MAPK1*, GRB2*, FOS*, SOS1*, MAPK3*,

Fc epsilon RI signaling pathway	7,22E-05	1	CSF2*, FCER1G*, PIK3CA*, PRKCD*, MAPK1*, FYN*, GRB2*, SOS1*, MAPK3*,
Small cell lung cancer	1,32E-04	1	CDK6*, CDKN1B*, CCND1*, PIK3CA*, ITGA2*, TRAF1*, PIAS2*, PTK2*, PIAS1*,
Long-term potentiation	2,16E-04	1	PPP3CA*, PPP3R1*, PPP3CC*, MAPK1*, CALM3*, CACNA1C*, GRIN2B*, MAPK3*,
Amyotrophic lateral sclerosis (ALS)	3,10E-04	1	PPP3CA*, PPP3R1*, PPP3CC*, CASP1*, GRIN2B*, MAP3K5*, TNFRSF1A*,
VEGF signaling pathway	4,00E-04	1	PPP3CA*, PPP3R1*, PPP3CC*, PIK3CA*, SRC*, MAPK1*, PTK2*, MAPK3*,
Non-homologous end-joining	9,15E-04	1	XRCC6*, PRKDC*,

From the threshold analysis at Figure 3, top-performing threshold value for the pooled dataset was 40%. Here we report Top20 PANOGA Functional Enrichment Results for genes having >40% methylation change. “Times Found” refers to number of genes included at our dataset which play role at that specific pathway. As a result, when the functional enrichment results of >15% and >40% are compared, >15% dominates in terms of correctly detecting thyroid-related pathways in top rankings.
